# *OGT* and *OGA* gene-edited human induced pluripotent stem cells for dissecting the functional roles of *O*-GlcNAcylation in hematopoiesis

**DOI:** 10.3389/fcell.2024.1361943

**Published:** 2024-05-01

**Authors:** Sudjit Luanpitpong, Kantpitchar Tangkiettrakul, Xing Kang, Pimonwan Srisook, Jirarat Poohadsuan, Parinya Samart, Phatchanat Klaihmon, Montira Janan, Chanchao Lorthongpanich, Chuti Laowtammathron, Surapol Issaragrisil

**Affiliations:** ^1^ Siriraj Center of Excellence for Stem Cell Research, Faculty of Medicine Siriraj Hospital, Mahidol University, Bangkok, Thailand; ^2^ Blood Products and Cellular Immunotherapy Research Group, Research Division, Faculty of Medicine Siriraj Hospital, Mahidol University, Bangkok, Thailand; ^3^ Division of Hematology, Department of Medicine, Faculty of Medicine Siriraj Hospital, Mahidol University, Bangkok, Thailand

**Keywords:** *O*-GlcNAcylation, induced pluripotent stem cell, hiPSC, hematopoietic stem cell, HSC, differentiation, *OGA*, *OGT*

## Abstract

Hematopoiesis continues throughout life to produce all types of blood cells from hematopoietic stem cells (HSCs). Metabolic state is a known regulator of HSC self-renewal and differentiation, but whether and how metabolic sensor *O*-GlcNAcylation, which can be modulated via an inhibition of its cycling enzymes *O*-GlcNAcase (OGA) and *O*-GlcNAc transferase (OGT), contributes to hematopoiesis remains largely unknown. Herein, isogenic, single-cell clones of *OGA*-depleted (OGAi) and *OGT*-depleted (OGTi) human induced pluripotent stem cells (hiPSCs) were successfully generated from the master hiPSC line MUSIi012-A, which were reprogrammed from CD34^+^ hematopoietic stem/progenitor cells (HSPCs) containing epigenetic memory. The established OGAi and OGTi hiPSCs exhibiting an increase or decrease in cellular *O*-GlcNAcylation concomitant with their loss of OGA and OGT, respectively, appeared normal in phenotype and karyotype, and retained pluripotency, although they may favor differentiation toward certain germ lineages. Upon hematopoietic differentiation through mesoderm induction and endothelial-to-hematopoietic transition, we found that OGA inhibition accelerates hiPSC commitment toward HSPCs and that disruption of *O*-GlcNAc homeostasis affects their commitment toward erythroid lineage. The differentiated HSPCs from all groups were capable of giving rise to all hematopoietic progenitors, thus confirming their functional characteristics. Altogether, the established single-cell clones of OGTi and OGAi hiPSCs represent a valuable platform for further dissecting the roles of *O*-GlcNAcylation in blood cell development at various stages and lineages of blood cells. The incomplete knockout of *OGA* and *OGT* in these hiPSCs makes them susceptible to additional manipulation, i.e., by small molecules, allowing the molecular dynamics studies of *O*-GlcNAcylation.

## Introduction

Hematopoiesis begins early in embryonic development and continues throughout life to give rise to all types of blood cells from hematopoietic stem cells (HSCs) through a series of progenitor stages with restricted lineage potentials (Rieger and Schroeder, 2012; Ito and Ito, 2018). HSC fates, including quiescence, self-renewal, lineage commitment and differentiation, migration or even apoptosis, are known to be regulated by various intrinsic and extrinsic signals, such as complex networks of transcription factors and signaling pathways, surrounding bone marrow microenvironment or niche, which is dynamic and typically altered in response to physiological cues, and interaction between the cells (Gautier et al., 2016; Goode et al., 2016; Fröbel et al., 2021). However, it is only recently that metabolic state has emerged as one of the key regulators of HSC self-renewal and differentiation. For example, the survival of quiescent HSCs in anaerobic bone marrow niche is dependent on active glycolysis, while oxidative phosphorylation (OXPHOS) promotes HSC differentiation (Suda et al., 2011; Morganti et al., 2022). It is also important to note the contributions of nutrients in lineage commitment. While utilization of glucose and glutamine fueling nucleotide biosynthesis enhances erythroid differentiation, blocking of glutamine favors the differentiation toward myelomonocytic lineage (Oburoglu et al., 2014; Oburoglu et al., 2016).

*O*-GlcNAcylation is an essential, ubiquitous posttranslational modification (PTM) that utilizes UDP-GlcNAc derived from amino acid, carbohydrate, fatty acid, and nucleotide metabolism in the hexosamine biosynthetic pathway (HBP) as a donor substrate to be added on serine/threonine residues of proteins by *O*-GlcNAc transferase (OGT) (de Queiroz et al., 2014; Ong et al., 2018). Its reverse reaction, *O*-GlcNAc hydrolysis, is catalyzed by *O*-GlcNAcase (OGA). The extent of *O*-GlcNAcylation generally reflects the global metabolic dynamics in the cells, and hence, it serves as an ideal metabolic sensor that participates in the regulation of various cellular processes through changes in protein function, gene expression, and cellular signaling. We previously reported that *O*-GlcNAcylation is a key determinant of human HSC fate decision and certain lineage-specific differentiation, including megakaryopoiesis (Luanpitpong et al., 2021), erythropoiesis (Luanpitpong et al., 2022a), and dendritic cell development (Luanpitpong et al., 2022b). Inhibition of OGT that causes a prolonged decrease in *O*-GlcNAcylation promotes megakaryopoiesis and subsequent thrombopoiesis through the perturbation of cell adhesion molecules. Meanwhile, inhibition of OGA was found to drive dendritic differentiation in both normal HSCs and malignant leukemic stem cells (LSCs) through STAT3/5 signaling. Significant gaps concerning the roles of *O*-GlcNAcylation in early HSC development and other stages of hematopoiesis remain largely unexplored.

Human induced pluripotent stem cells (hiPSCs), which have the capacity for self-renewal and differentiation into most, if not all, somatic cells, offer an experimental tool for studying hematopoiesis as they could recapitulate early events in human blood development (Georgomanoli and Papapetrou, 2019; Pratumkaew et al., 2021). In the present study, we generated isogenic, single-cell clones of *OGA*-depleted (OGAi) and *OGT*-depleted (OGTi) hiPSCs using CRISPR/Cas9 system and performed thorough hiPSC characterization. The hiPSC line MUSIi012-A, which was reprogrammed from G-CSF mobilized peripheral blood stem cells of a healthy donor (Laowtammathron et al., 2019), was chosen as the parental cells as they may contain epigenetic memory that predisposes the cells to differentiation into HSCs and other blood cells (Kim et al., 2010; Poetsch et al., 2022). A better insight into the regulation of hematopoiesis would enable the efficient production of different blood lineages from HSCs from various sources, including renewable hiPSCs, which may benefit the translation into future clinical applications (Arias et al., 2021; Sugimoto et al., 2022). The established OGTi and OGAi hiPSCs can be used as a platform for dissecting the functional roles of metabolic sensor *O*-GlcNAcylation in early HSC development and various stages of hematopoiesis. Our initial finding herein, for the first time, highlighted the impact of *O*-GlcNAcylation during HSC differentiation from hiPSCs through mesoderm induction and endothelial-to-hematopoietic transition (EHT).

## Methods

### Materials

Full details of key resources can be found in Supplementary Table S1.

### Cell culture

The parental hiPSC line, registered in the Human Pluripotent Stem Cell Registry (hPSCreg^®^) as MUSIi012-A (accessible at https://hpscreg.eu/cell-line/MUSIi012-A), was established from CD34^+^ hematopoietic stem/progenitor cells (HSPCs) collected from G-CSF mobilized peripheral blood of a healthy female donor using non-integrating episomal reprogramming plasmids under ethics approval by Siriraj Institutional Review Board (COA No. Si 248/2011) after informed consent. Cells were maintained on Matrigel-coated plates (Corning, Corning, New York, United States) in NutriStem XF medium (Sartorius, Göttingen, Germany), passaged every 2–3 days using Versene solution (Gibco, Waltham, MA, United States), and cultured in a humidified atmosphere of 5% CO_2_ at 37°C.

### CRISPR/Cas9-mediated gene knockdown

**FIGURE 1:**
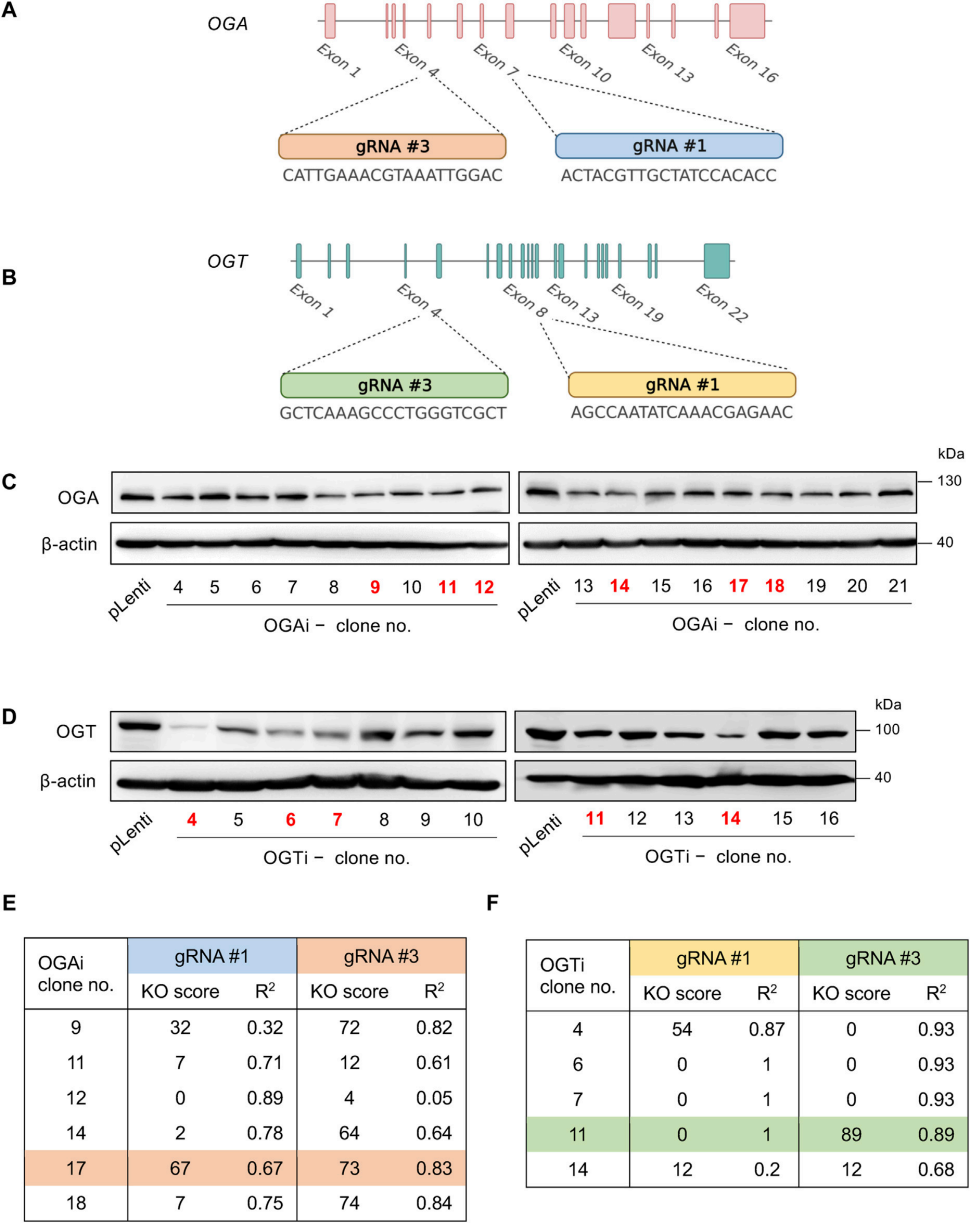
Single-cell cloning of *OGA*- and *OGT*-depleted hiPSCs. **(A, B)** Strategy to target *OGA* **(A)** and *OGT* **(B)** using CRISPR/Cas9 system with two gRNAs (#1 and #3), generating OGAi and OGTi hiPSCs, respectively. **(C)** Western blot analysis for screening of OGAi hiPSC single-cell clones with abolished OGA level. Clone numbers 9, 11, 12, 14, 17, and 18 were selected for further genomic DNA sequencing and ICE analysis. **(D)** Western blot analysis for screening of OGTi hiPSC clones with abolished OGT level. Clone numbers 4, 6, 7, 11, and 14 were selected for further analyses. **(E, F)** Summary of KO scores determined by ICE in the DNA sequencing spanning over Cas9 cut sites on *OGA* **(E)** and *OGT* **(F)** guided by corresponding gRNA #1 and #3 in OGAi and OGTi hiPSCs, respectively.

All-in-one lentiviral plasmids (pLentiCRISPR v2) carrying SpCas9, puromycin resistance, and guide RNAs (gRNAs) against human *OGA* (also known as *MGEA5*) and *OGT* were obtained from GenScript (Piscataway, New Jersey, United States). For better knockdown efficiency, we used two gRNAs each to target *OGA* and *OGT*—the oligo sequences of all gRNAs and editing strategy are schematically shown in Figures 1A, B. Lentiviral production was performed using HEK293FT packaging cells (American Type Culture Collection, ATCC; Manassas, VA) in conjunction with pCMV.dR8.2 dvpr lentiviral packaging and pCMV-VSV-G envelope plasmids (Addgene #8454 and #8455) (Stewart et al., 2003). Parental MUSIi012-A hiPSCs were dissociated into a single cell suspension using Accutase (STEMCELL Technologies, Vancouver, BC, Canada) before seeding onto a Matrigel-coated plate for 24 h and sequentially transduced with concentrated lentiviral particles in the presence of hexadimethrine bromide (8 μg/mL) for 48 h. After which, the cells were treated with 2 μg/mL puromycin for 2 days to eliminate the non-transduced cells, and the remaining cells were subjected to single-cell cloning.

### Single-cell cloning of isogenic OGAi and OGTi hiPSCs

A single-cell suspension of transduced hiPSCs was prepared in Nutristem XF medium at a density of 20 cells/mL, and loaded into a Matrigel-coated 96-well plate. The presence of a single cell in each well was confirmed under an inverted microscope, and mouse embryonic fibroblasts (MEFs) were seeded into each well on top of the single cells. Emerging colonies were mechanically picked up and expanded in a Matrigel-coated plate for further screening for the successful gene knockdown by Western blotting and genomic DNA sequencing.

### Western blot analysis

Cells were harvested and lysed by incubation with lysis buffer (Cell Signaling Technology, Denvers, MA) containing protease inhibitors (Roche Diagnostics, Mannheim, Germany) at 4°C for 30 min. Approximately 50 μg of proteins as determined by a BCA protein assay (Pierce Biotechnology, Waltham, MA, United States) was subjected to SDS-PAGE and transferred onto PVDF membranes. The membranes were blocked with 5% skim milk, and incubated with appropriate primary antibodies at 4°C overnight and HRP-conjugated secondary antibodies for 1 h at room temperature. The immune complexes were analyzed by an enhanced chemiluminescence detection system (Merck Millipore, Burlington, MA) on a digital imager ImageQuant LAS (GE Healthcare, Chicago, IL, United States).

### Genomic DNA sequencing and INDEL analysis

Genomic DNA was isolated using a PureLink Genomic DNA Mini Kit (Invitrogen, Waltham, MA, United States). The target regions for DNA sequencing were amplified by polymerase chain reaction (PCR) using Q5 High-Fidelity DNA Polymerase (New England Biolabs, Ipswich, MA, United States) with specific primers, and the resulting PCR products were purified by a GenepHlow Gel/PCR kit (Geneaid, New Taipei City, Taiwan). A total of 0.2 μg of PCR product was then used for DNA sequencing using ABI PRISM BigDye Terminator Cycle Sequencing Kit v3.1 (first BASE, Singapore). The presence of insertion/deletion (INDEL) mutations in each target gene was determined by Inference of CRISPR Edits (ICE), the web-based analysis tool (available at https://ic-e.synthego.com/). Knockout (KO) score, which represents the proportion of cells that have either a frameshift or 21+ bp INDEL that are likely to generate a complete loss-of-function mutation, was determined.

### CRISPR off-target analysis

The potential off-target for each gRNA was predicted by CRISPR/Cas9 target online predictor (CCTop; available at http://crispr.cos.uni-heidelberg.de) (Stemmer et al., 2015). The top five off-target sequences of each gRNA were amplified and checked by genomic DNA sequencing to confirm no mutations.

### Immunofluorescence staining

Cells were fixed in 4% paraformaldehyde for 20 min, permeabilized with 0.1% Triton X-100/PBS for 10 min, and blocked with 3% bovine serum albumin (BSA)/PBS for 1 h. The cells were incubated with primary antibodies in 1% BSA/PBS overnight at 4°C, followed by Alexa Fluor 488 secondary antibodies at room temperature for 1 h. After which, they were visualized under fluorescence microscope (Eclipse Ti-U, Nikon, Tokyo, Japan) with NIS-Elements D Software (version 4.30.00; Nikon).

### Flow cytometry

For analysis of pluripotency markers, hiPSCs were dissociated into single cells using TrypLETM Select (Gibco), blocked with 10% human AB serum, stained with FITC-conjugated SSEA-3, PE-conjugated SSEA-4, and Alexa Fluor 647- conjugated TRA-1-60 (BioLegend, San Diego, CA, United States), and analyzed by BD FACS Canto (BD Biosciences, Franklin Lakes, NJ, United States).

For immunophenotypic analysis of the differentiated HSPCs, cells were resuspended in FACS buffer (PBS with 2% BSA), incubated with antibody cocktail for 30 min at 4°C in the dark, and analyzed by BD FACS Canto. The antibodies used in this study included FITC-conjugated CD34, PerCP-conjugated CD45, PE-Cy7-conjugated CD43 (BD Biosciences), and APC-conjugated CD235a (glycophorin A, GPA) (BioLegend). Absolute counts were performed using BD Trucount tubes containing a known quantity of fluorescent beads.

### Quantitative PCR analysis

Total RNA was isolated using TRI Reagent^®^ (Molecular Research Center, Cincinnati, OH, United States) and converted to complementary DNA using the RevertAid First Strand cDNA synthesis kit (Thermo Fisher Scientific, Waltham, MA, United States). The quantitative PCR (qPCR) reactions were performed on the CFX384 Touch Real-Time PCR detection system (Bio-Rad, Hercules, CA, United States) using SYBR™ Select Master Mix (Thermo Fisher Scientific). The oligonucleotide sequences of forward and reverse primers used can be found in Supplementary Table S2. The cycle parameters started with an activation step at 95°C for 2 min, followed by 40 cycles of denaturation at 95°C for 15 s and annealing/extension at 60°C for 1 min.

### Karyotyping

Standard G-banded karyotyping was performed at the Department of Obstetrics and Gynecology, Faculty of Medicine Siriraj Hospital, Mahidol University. A total of 25 metaphases at a band resolution of 400–450 were analyzed.

### Short tandem repeat analysis

Short tandem repeat (STR) analysis was performed at the Department of Forensic Medicine, Faculty of Medicine Siriraj Hospital, Mahidol University. A total of 16 loci were tested.

### Spontaneous *in vitro* differentiation via embryoid body formation

hiPSCs were harvested into small clumps using 1 mg/mL Dispase (Gibco) and cultured on low attachment dishes in KnockOut™ DMEM supplemented with 20% KnockOut™ Serum Replacement, 2 mM GlutaMAX™, 0.1 mM MEM non-essential amino acid, 0.1 mM β-mercaptoethanol, 1X insulin-transferrin-selenium-ethanolamine, and 100 U/mL penicillin/streptomycin (Gibco). The medium was replaced every other day. On day 7, embryoid bodies (EBs) were transferred onto a 0.1% gelatin-coated plate and cultured at 37°C and 5% CO_2_ for another 3 weeks.

### Differentiation of hiPSCs into HSPCs

hiPSCs were pretreated with Rho kinase (ROCK) inhibitor Y27632 for 1 h before single cell dissociation by Accutase™ (Gibco) and plated into an AggreWell™ 800, 24-well plate at a density of 2.16×10^6^ cells/well in basal differentiation medium (DM), which was StemPro™-34 medium containing 2 mM GlutaMAX™, 0.4 mM monothioglycerol (MTG), 150 μg/mL transferrin, and 50 μg/mL L-ascorbic acid, supplemented with 10 ng/mL recombinant human (rh) BMP4 and 10 μM ROCK inhibitor. Cells were cultured under a hypoxic environment containing 5% O_2_ and 5% CO_2_ at 37°C. The EBs that arose on the following day were transferred to a 6-well plate and cultured in DM supplemented with 10 ng/mL rhBMP4, 5 ng/mL rh bFGF, and 3 μM CHIR99021. On day 3 of culture, the EBs were transferred to Matrigel-coated plates and cultured in DM supplemented with 5 ng/mL rh bFGF, 15 ng/mL rh VEGF, 30 ng/mL rh IL-3, 10 ng/mL rh IL-6, 5 ng/mL rh IL-11, 25 ng/mL rh IGF-1, 50 ng/mL rh SCF, 2 U/mL rh erythropoietin (EPO), 30 ng/mL rh thrombopoietin (TPO), and 10 ng/mL rh Flt-3L. On day 6 of culture onwards, an extra 10 ng/mL rh BMP4 was added to the culture. Differentiated cells were changed back to be maintained under a normoxic environment containing 5% O_2_ on day 8 of culture.

### Colony-forming unit assay

Colony-forming unit (CFU) assay was performed using enriched methylcellulose (MC) medium (MethoCult H4435 Enriched; STEMCELL Technologies), which supports the growth of multipotential progenitor cells, including CFU-granulocyte, erythrocyte, macrophage, megakaryocyte (CFU-GEMM), granulocyte and macrophage progenitor cells (CFU-granulocyte, macrophage (CFU-GM)), CFU-macrophage (CFU-M), and erythroid progenitor cells (burst-forming unit-erythroid (BFU-E) and CFU-erythroid (CFU-E)), according to the manufacturer’s instructions. Briefly, 8,000 starting cells were gently dispersed in the enriched MC, seeded onto 12-well plates, and cultured for 14 days. Colonies were identified and counted in a double-blind manner by two certified medical technicians under an inverted microscope (Eclipse Ti-U, Nikon, Tokyo, Japan).

### Statistical analysis

Data are presented as mean ± SD from three or more independent experiments. Statistical analysis was performed by one-way ANOVA with Tukey’s multiple comparison test at a significance level of *p* < 0.05 using GraphPad Prism software (San Jose, CA, United States).

## Results

### CRISPR/Cas9-mediated gene knockdown of *OGA* and *OGT* in hiPSCs

**FIGURE 2:**
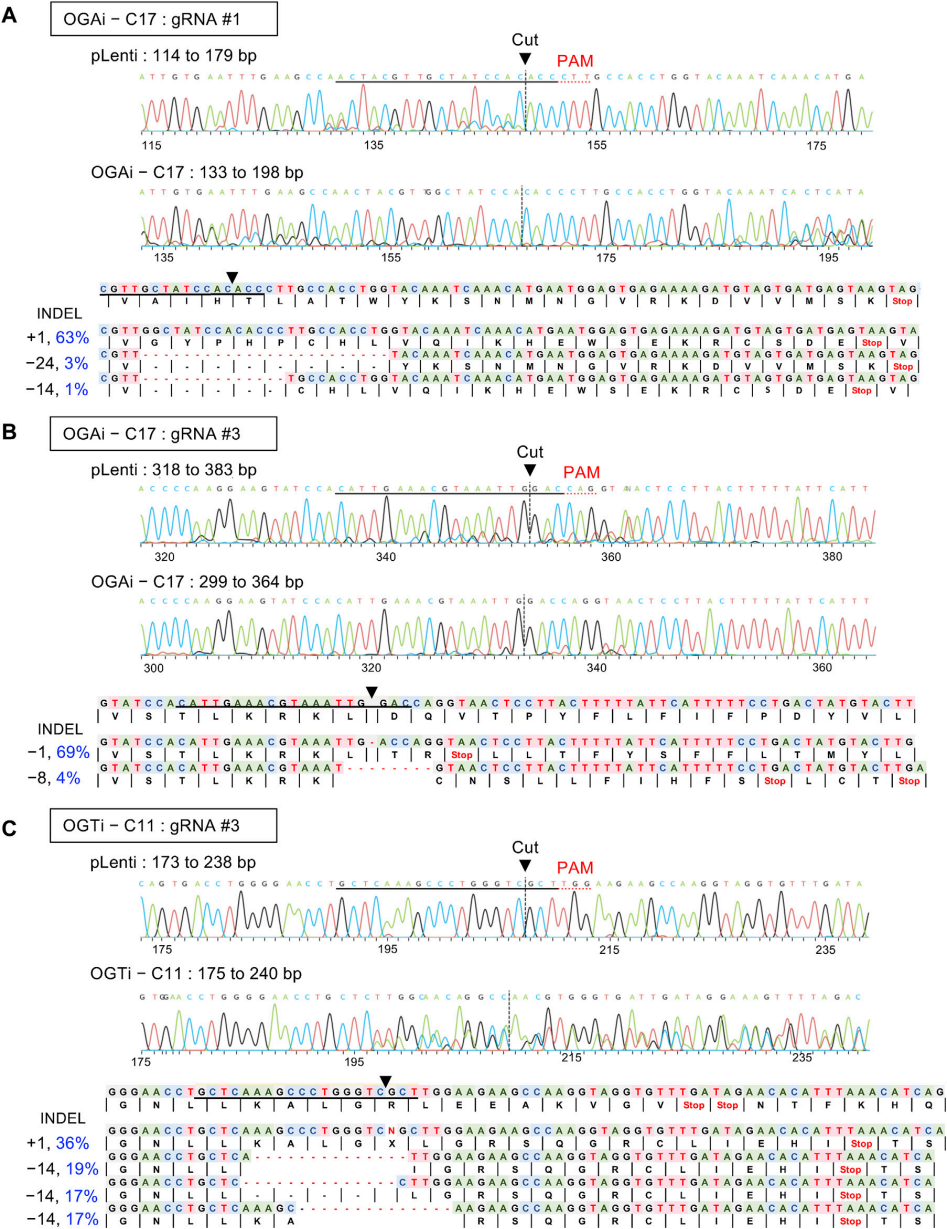
Characterization of INDEL mutations in OGAi and OGTi hiPSCs by DNA sequencing and ICE analysis. **(A, B)** (upper) DNA sequencing spanning over the Cas9 cut sites on *OGA* when using gRNA #1 **(A)** and #3 **(B)** from CRISPR control (pLenti) hiPSCs in comparison with OGAi–C17 hiPSCs. The gRNA sequence is underlined in black, and the PAM sequence is denoted by a dotted red underline in the control sample. Vertical dotted lines denote the expected cut site. (lower) The inferred sequences of *OGA* present in OGAi–C17 hiPSCs and their relative proportions (%INDEL) are listed along with the translated amino acids. **(C)** (upper) DNA sequencing spanning over the Cas9 cut site on *OGT* when using gRNA #3 from CRISPR control (pLenti) hiPSCs in comparison with OGTi–C11 hiPSCs. (lower) The inferred sequences of *OGT* present in OGTi–C11 hiPSCs and their relative proportions (%INDEL) are listed along with the translated amino acids.

To determine the functional relationship between the cellular *O*-GlcNAc level and hematopoiesis at various stages, we used the CRISPR/Cas9 system to deplete *OGA* and *OGT* expression in MUSIi012-A hiPSCs using two gRNAs to target each gene to improve the editing efficiency and generate a complete loss-of-function mutation, as schematically depicted in Figures 1A, B. The cells were sequentially transduced with lentiviral particles comprising gRNA #1 and #3 against *OGA* (OGAi) or *OGT* (OGTi) and SpCas9, subjected to puromycin selection, and single-cell cloning. Figures 1C, D show the OGA and OGT levels in different clones of OGAi and OGTi hiPSCs that emerged from single-cell cloning in comparison to their CRISPR vector control (pLenti) hiPSCs. Next, OGAi hiPSCs with decreased OGA level, including clone numbers 9, 11, 12, 14, 17, and 18 were subjected to genomic DNA sequencing and subsequent ICE analysis, which could examine the presence of INDEL mutations and predict gene editing efficiency by means of KO score. The KO scores of each gRNA targeting *OGA* in each hiPSC clone are summarized in Figure 1E. OGAi clone 17 (C17) hiPSCs were eventually chosen for further studies, as it had the highest KO score, indicating the best functional knockout. Likewise, OGTi hiPSCs clone numbers 4, 6, 7, 11, and 14 with decreased OGT level were subjected to genomic DNA sequencing and ICE analysis, and clone 11 (C11) was chosen (Figure 1F). Figures 2A, B show the DNA sequencing spanning over the Cas9 cut sites on *OGA* when using gRNA #1 and #3 in OGAi–C17 hiPSCs, respectively. All major frameshifts created stop codons but the early stop codons were created by gRNA #3, which caused a −1 bp deletion. For OGTi–C17 hiPSCs, only gRNA #3 caused INDEL mutations, as validated by DNA sequencing spanning over its Cas9 cut site on *OGT* in Figure 2C. We also performed DNA sequencing spanning over the potential Cas9 cut sites on off-target genes of each gRNA, as predicted by the CCTop tool (see Supplementary Figure S1), to ensure no potential off-targets.

### Modulation of cellular *O*-GlcNAc level in OGAi and OGTi hiPSCs

**FIGURE 3:**
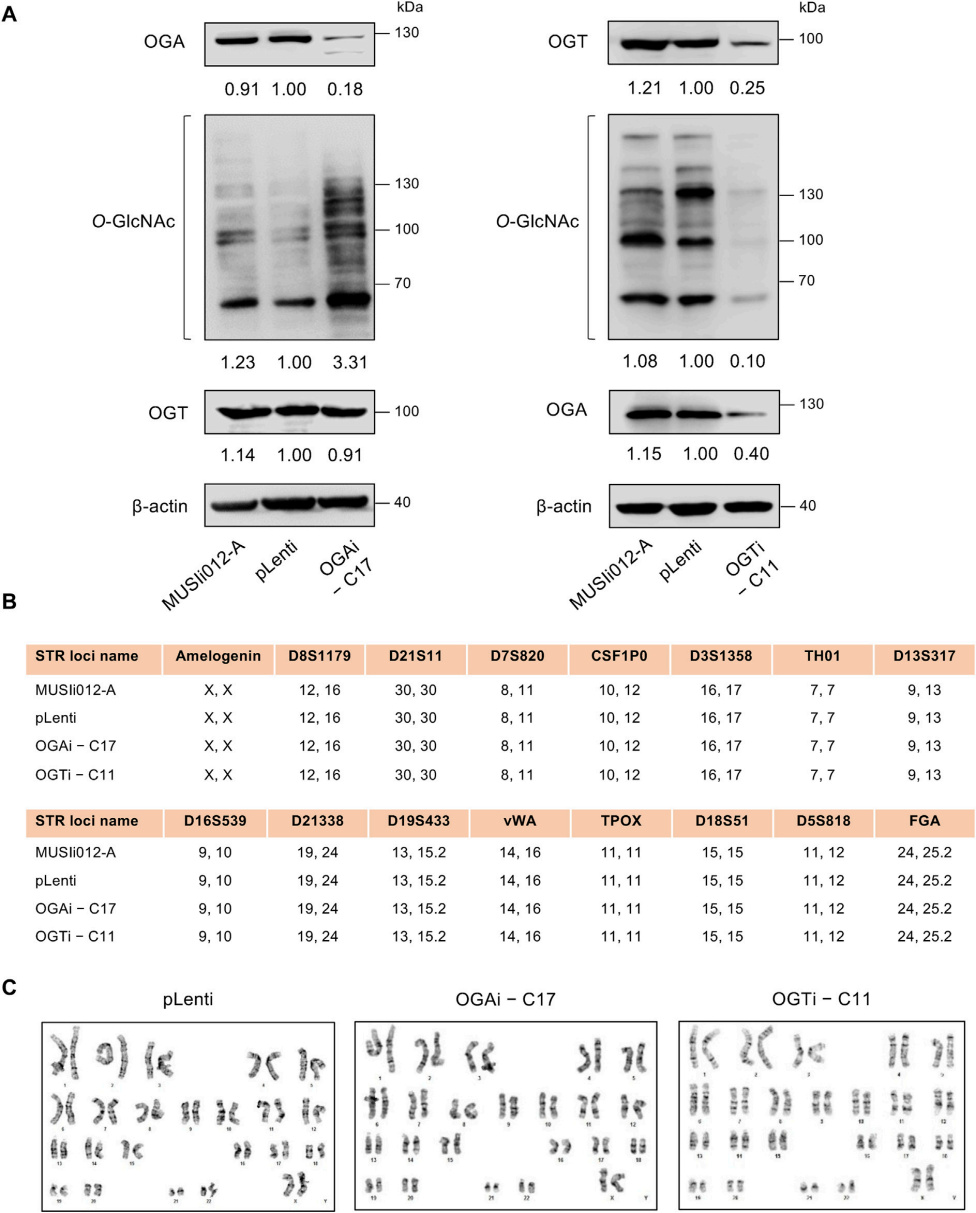
Modulation of cellular O-GlcNAcylation in OGAi and OGTi hiPSCs. **(A)** Western blot analysis of OGA, OGT, and O-GlcNAc levels showing knockdown efficiency and modulation of *O*-GlcNAcylation in OGAi–C17 (left) and OGTi–C11 (right) hiPSCs in comparison with CRISPR control (pLenti) hiPSCs. Blots were reprobed with anti-β-actin antibody to establish a loading control. **(B)** STR analysis comparing a total of 16 loci between the parental MUSIi012-A and the newly established pLenti, OGAi–C17, and OGTi–C11 hiPSCs. **(C)** Karyotype analysis as determined by G-banding assay.

Having demonstrated the deletion of *OGA* and *OGT* in OGAi–C17 and OGTi–C11 hiPSCs, respectively, we evaluated the cellular *O*-GlcNAc level in these cells to ascertain the successful alteration of PTM of total proteins. Figure 3A shows a significant increase in *O*-GlcNAc level in OGAi–C17 hiPSCs when compared to its counterpart pLenti control that was concomitant with the loss of OGA. Conversely, *O*-GlcNAc level was barely detected in OGTi–C11 hiPSCs. A decrease in OGA level was observed in OGTi–C11 hiPSCs, which was not totally unexpected. Numerous studies have demonstrated that OGA level decreases when *O*-GlcNAcylation is blocked, e.g., by small molecule inhibitors and substrate mimics (Gloster et al., 2011; Ortiz-Meoz et al., 2015). STR analyses confirmed the isogenic identity of OGAi–C17, OGTi–C11, and pLenti hiPSCs to their parental cell line MUSIi012-A (Figure 3B). These cells also exhibited stable diploid karyotypes (46, XX) at 400–500 resolution after being expanded for >20 passages from single-cell cloning (Figure 3C). Basal cell death rates, as evaluated by Annexin V/7-AAD assay, were not significantly different among groups (Supplementary Figure S2).

### Characterization of OGAi and OGTi hiPSCs and their multilineage differentiation

**FIGURE 4:**
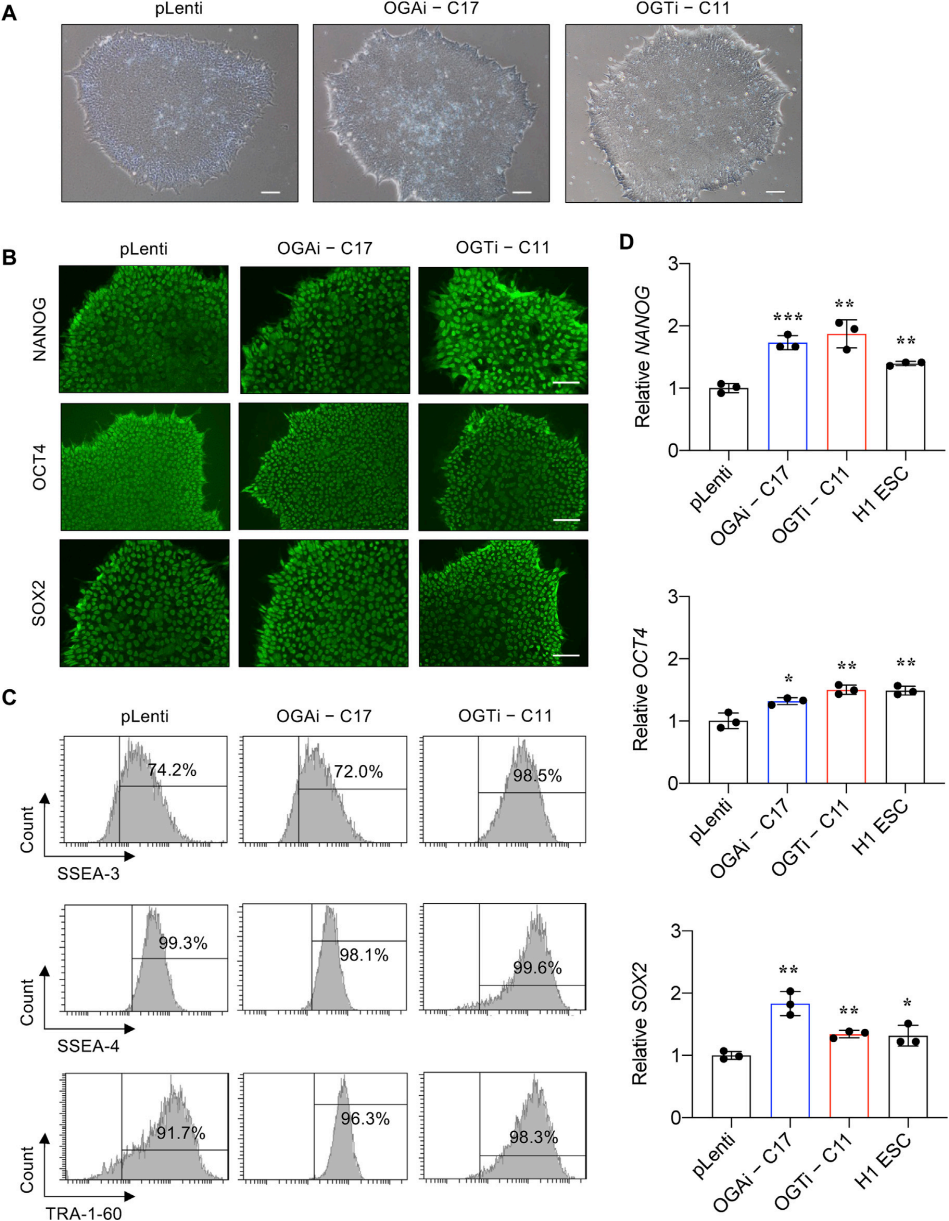
Characterization of an established single-cell clone of OGAi and OGTi hiPSCs. **(A)** Representative micrographs of pLenti, OGAi–C17, and OGTi–C11 hiPSC colonies. **(B)** Representative micrographs of fluorescence staining showing the pluripotency markers NANOG, OCT4, and SOX2. **(C)** Flow cytometric analysis of pluripotency markers SSEA-3, SSEA-4, and TRA-1-60. Marker bound over the histogram defines the positive cells. **(D)** mRNA expression of *NANOG*, *OCT4*, and *SOX2* by qPCR. Data are mean ± SD (n = 3). ^*^ *p* < 0.05, ^**^ *p* < 0.01, ^***^ *p* < 0.001 *versus* pLenti; unpaired *t*-test. H1 ESCs were used as the positive control cells for pluripotency.

We next validated that OGAi–C17 and OGTi–C11 hiPSCs retained their pluripotency by first visualizing their gross morphology, which is an important signature to monitor the culture status (Nagasaka et al., 2017). The morphological criteria of undifferentiated hiPSCs, so-called embryonic stem cell (ESC)-like colony, are i) relatively round colony with distinct border and well-defined edge and ii) tightly packed cells with a high nucleus-to-cytoplasm ratio (Wakao et al., 2012). Figure 4A shows that OGAi–C17, OGTi–C11, and pLenti hiPSCs exhibited the typical morphology of healthy, undifferentiated hiPSCs with a dense colony center. The presence of well-characterized and widely accepted pluripotency markers was evaluated using immunofluorescence (NANOG, OCT4, and SOX2) (Figure 4B), flow cytometry (SSEA-3, SSEA-4, and TRA-1-60) (Figure 4C), and qPCR (*NANOG*, *OCT4*, and *SOX2*) in comparison to the well-established H1 hESC cell line (Figure 4D).

**FIGURE 5:**
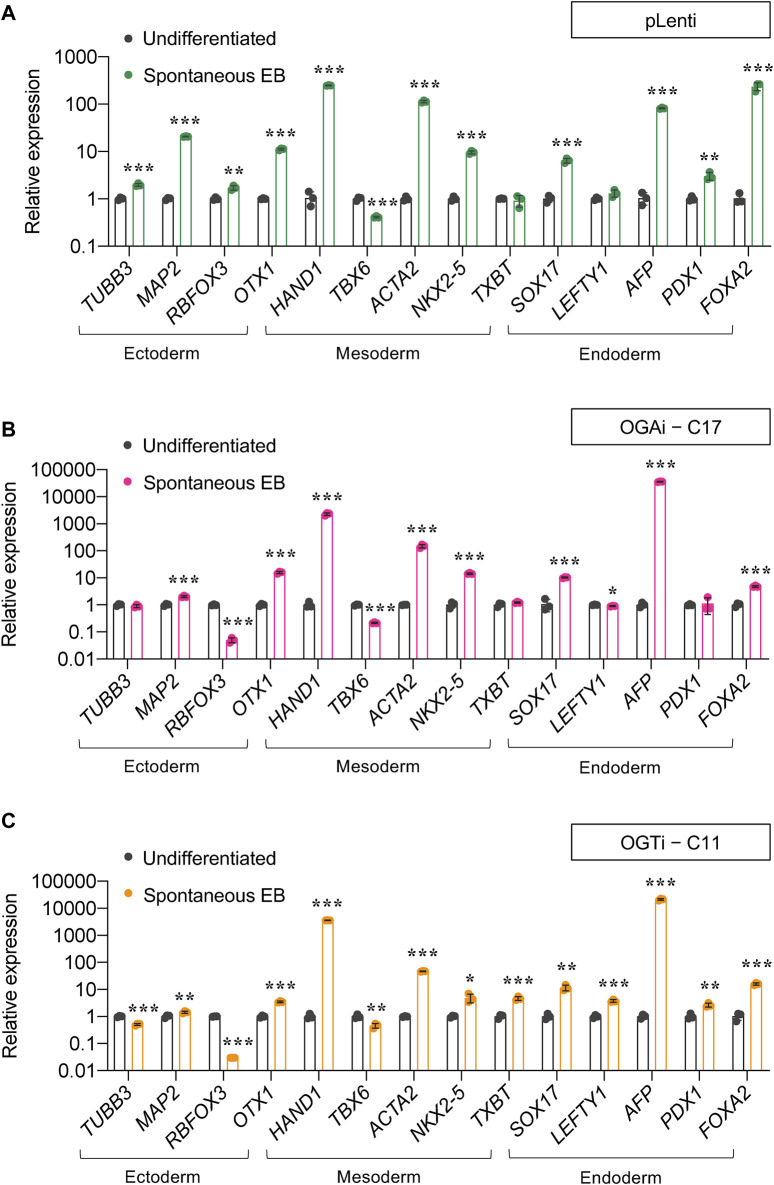
Differentiation potential of OGAi and OGTi hiPSCs as evaluated by spontaneous EB formation. **(A–C)** mRNA expression of trilineage differentiation markers: *TUBB3*, *MAP2*, *RBFOX3*, and *OTX1* for ectoderm; *HAND1*, *TBX6*, *ACTA2*, and *TBXT* for mesoderm; and *SOX17*, *LEFTY1*, *AFP*, *PDX1*, and *FOXA2* for endoderm in EBs derived from pLenti **(A)**, OGAi–C17 **(B)**, and OGTi–C11 **(C)** hiPSCs on day 21 of differentiation by qPCR. Data are mean ± SD (n = 3). ^*^ *p* < 0.05, ^**^ *p* < 0.01, ^***^ *p* < 0.001 *versus* undifferentiated hiPSCs; unpaired *t*-test.

For a functional assay on the capability of the cells to differentiate into ectoderm, endoderm, and mesoderm, we utilized spontaneous *in vitro* differentiation by EB formation. Figures 5A–C show a remarkable upregulation of two or more established derivatives’ markers for ectoderm (*TUBB2*, *MAP2*, *RBFOX3*, and *OTX1*), mesoderm (*HAND1*, *TBX6*, *ACTA2*, and *NKX2.5*), and endoderm (*SOX17*, *LEFTY1*, *AFP*, and *FOXA2*) in EBs derived from OGAi–C17, OGTi–C11, and pLenti hiPSCs on day 14 of differentiation when compared to undifferentiated cells, thus confirming their multilineage differentiation potential. It is worth noting that OGAi–C17 and OGTi–C11 hiPSCs expressed a relatively high expression of mesoderm gene *HAND1* (>2,000-fold *versus* 250-fold in pLenti hiPSCs) and endoderm gene *AFP* (>20,000-fold *versus* 80-fold in pLenti hiPSCs), while they expressed a strikingly low expression of ectoderm gene *RBFOX3*. Altogether, these data indicate that manipulation of cellular *O*-GlcNAcylation in hiPSCs via genetic inhibition of OGA and OGT, although it did not lead to the loss of general pluripotency, may favor the differentiation toward certain lineages.

### Hyper-*O*-GlcNAcylation promotes hematopoietic commitment of hiPSCs

**FIGURE 6:**
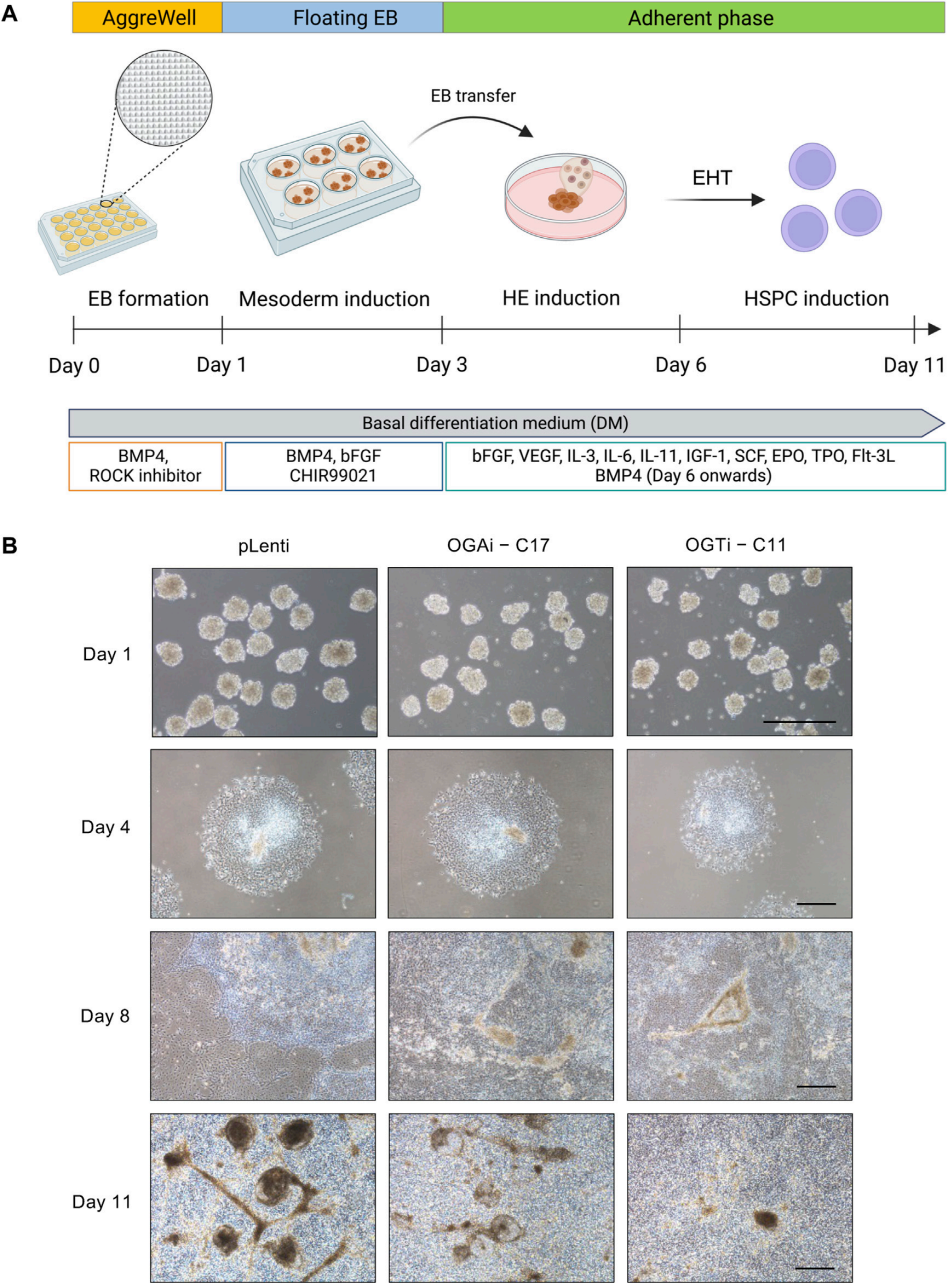
Generation of floating EBs and HSPC induction from hiPSCs. **(A)** Schematic diagram showing the two-stage differentiation protocol using defined differentiation medium. **(B)** Morphology of generated floating EBs on day 1 and adherent EBs on day 4 of culture. Floating hematopoietic cells from the adherent EBs were observed on day 8 of culture onwards. Scale bar = 400 μm.

hiPSCs hold great promise for cell-based therapy, including cancer immunotherapy. The differentiation of anti-BCMA CAR-natural killer (NK) cells (FT576) from CD38-knockout hiPSCs that expressed anti-BCMA chimeric antigen receptor (CAR), high-affinity, non-cleavable CD16 (hnCD16), and IL-15/IL-15 receptor fusion protein (IL-15RF) is currently undergoing Phase 1 clinical trial (NCT 05182073) for the treatment of multiple myeloma (Wang X. et al., 2022). Generally, the differentiation of hiPSCs into any specialized blood cells begins with the induction of hiPSCs into mesoderm and hemogenic endothelium (HE) to generate HSPCs, which recapitulates the development of HSCs during hematopoiesis (Pratumkaew et al., 2021; Klaihmon et al., 2023). Herein, we investigated the impact of *O*-GlcNAcylation on hiPSC commitment to hematopoietic lineage using a differentiation protocol consisting of i) mesoderm induction via AggreWell EB formation and ii) HSPC induction via EHT, as schematically described in Figure 6A. The morphology of adherent EBs on day 4 in a Matrigel-coated plate was considered healthy, as they maintained a round morphology and exhibited continued outgrowth (Figure 6B). On day 8 of culture, the formation of cobblestone areas and round-shaped floating cells were clearly observed. The switch of culture from adherent cells to floating cells is essential for undergoing EHT (Wang N. et al., 2022). Hence, the floating cells were collected on day 8 onwards for further immunophenotypic analyses by flow cytometry.

**FIGURE 7:**
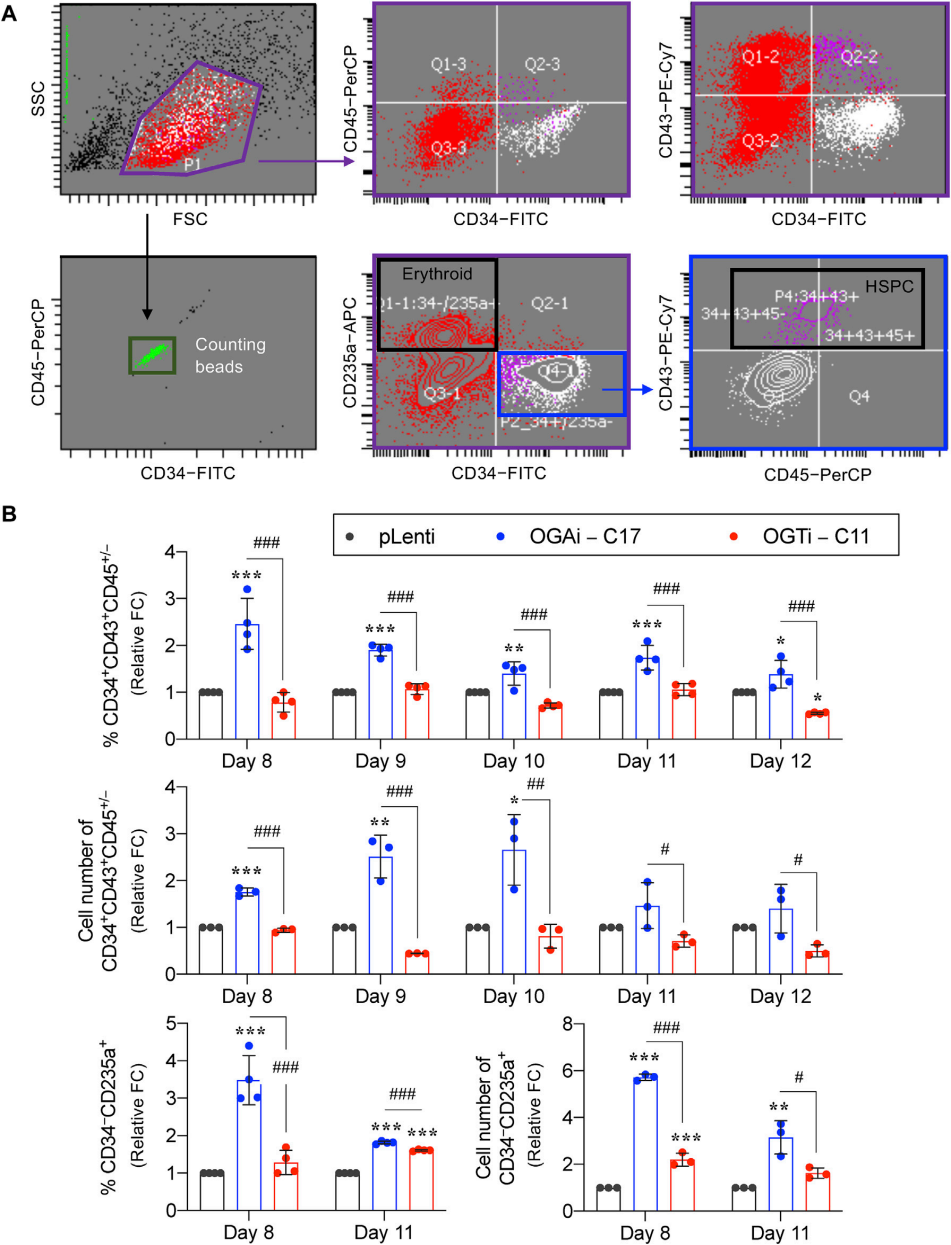
Cellular *O*-GlcNAcylation affects hiPSC differentiation toward HSPCs. **(A)** Flow cytometry gating strategy to define floating cells derived from pLenti, OGAi–C17, and OGTi–C11 hiPSCs into CD34^+^CD43^+^CD45^+/−^ HSPCs and CD34^−^CD235a^+^ erythroid cells. Absolute cell numbers were determined by comparing cell events to counting bead events. **(B)** Percentage (upper) and cell number (middle) of derived CD34^+^CD43^+^CD45^+/−^ HSPCs on days 8–12 of culture. (lower) Percentage and cell number of derived CD34^−^CD235a^+^ cells on days 8 and 11 of culture. Due to inter-experimental variations, data are presented as relative fold change (FC) to pLenti control in each experiment. Data are mean ± SD (*n* = 3 or 4) from at least three independent experiments. ^*^ *p* < 0.05, ^**^ *p* < 0.01, ^***^ *p* < 0.001 *versus* pLenti control group; ^#^ *p* < 0.05, ^##^ *p* < 0.01, ^###^ *p* < 0.001 *versus* OGAi group; one-way ANOVA with Tukey’s multiple comparison test. Representative flow cytometry plots can be found in Supplementary Figures S3, S4.

HSPCs were defined as CD34^+^CD43^+^CD45^+/−^ cells. As the red color of cell pellets was noticeable, especially in cells derived from OGAi–C17 and OGTi–C11 hiPSCs, we also evaluated for CD34^−^CD235a^+^ erythroid cells. Flow cytometry gating strategy is shown in Figure 7A. Figure 7B shows that OGAi–C17 hiPSCs generated a significantly higher percentage of HSPCs than pLenti hiPSCs on day 8 (approximately 2.5-fold), which remained relatively higher toward the end of culture. Conversely, the percentage of HSPCs in OGTi–C11 hiPSCs was observed to be lower than that in OGAi–C17 hiPSCs. Consistently, the absolute cell number of HSPCs derived from OGAi–C17 hiPSCs was remarkably greater than those from pLenti and OGTi–C11 hiPSCs, particularly on days 9 and 10 of culture, with an approximately 3-fold increase when compared with pLenti hiPSCs, indicating that OGA inhibition accelerates the hiPSC commitment toward HSPCs. We also found an increase in percentage and cell number of CD235a^+^ erythroid cells upon inhibition of OGA and OGT, suggesting that *O*-GlcNAc homeostasis plays an important role in erythroid commitment of hiPSCs.

### HSPCs derived from OGAi hiPSCs display normal function

**FIGURE 8:**
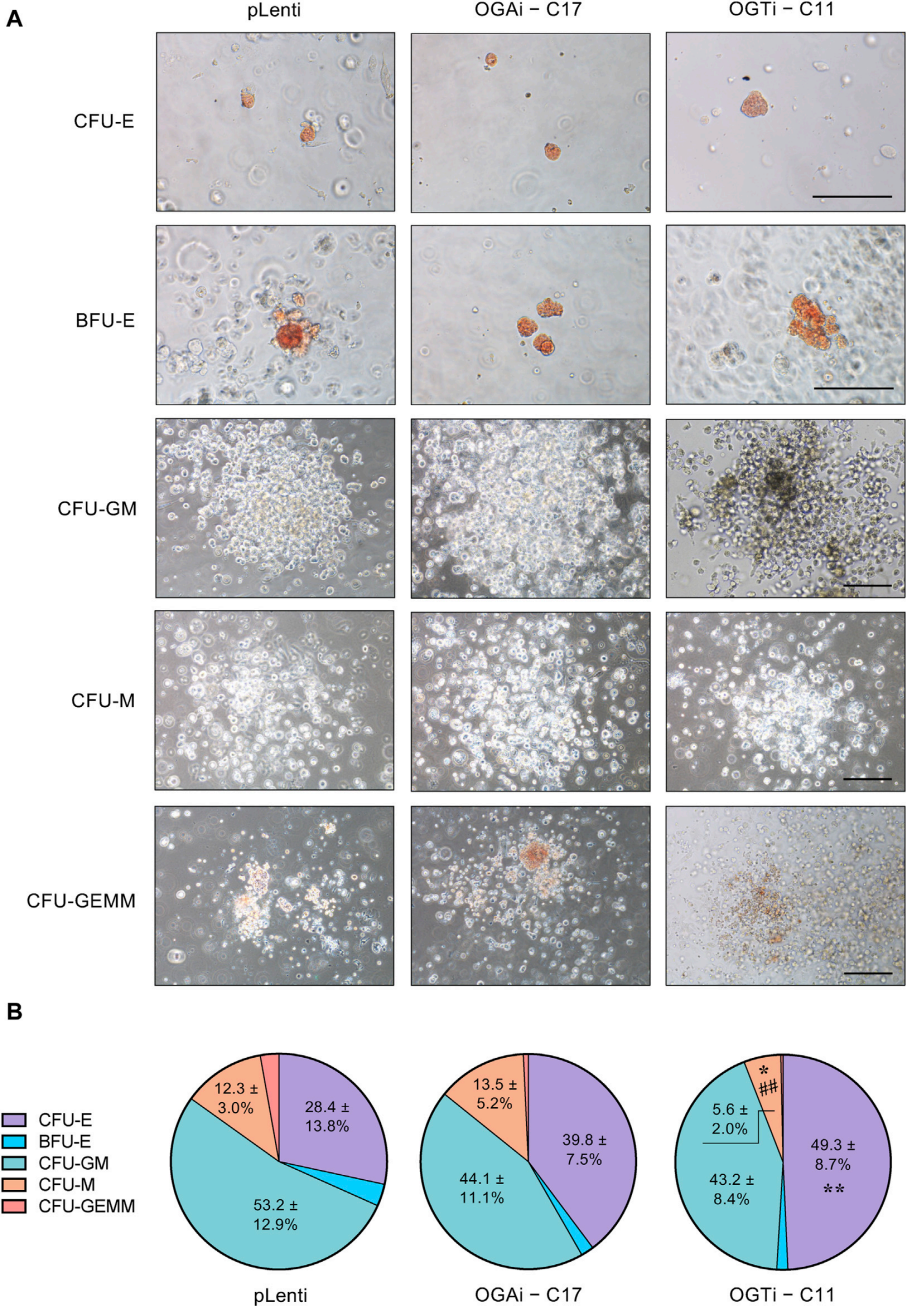
Functional characterization of the differentiated HSPCs from hiPSCs. **(A)** Representative micrographs of different progenitor cell colonies growing from CFU assay. HSPCs derived from pLenti, OGAi–C17, and OGTi–C11 hiPSCs at day 11 of culture were used. Scale bar = 200 μm. **(B)** Colonies were scored under an inverted microscope at 14 days of culture and the proportion of each progenitor was plotted. Data are mean ± S.D. (*n* = 6) from six independent experiments. ^*^ *p* < 0.05, ^**^ *p* < 0.01 *versus* pLenti control group; ^##^ *p* < 0.01 *versus* OGAi group; one-way ANOVA with Tukey’s multiple comparison test. See also Supplementary Table S3 for average number of CFU colonies counted.

Having demonstrated that OGAi–C17 hiPSCs yielded the best outcome for HSPCs, the existence of functionally active HSPCs was verified based on its differentiation capacity into different hematopoietic progenitors. Differentiated HSPCs were enriched from OGAi–C17, OGTi–C11, and pLenti hiPSCs in specialized DM on day 11 of culture and subjected to CFU differentiation assay. Figure 8A shows that the differentiated HSPCs from all groups were able to generate erythroid, myeloid, and mixed hematopoietic progenitor colonies, including CFU-E, BFU-E, CFU-M, CFU-GM, and CFU-GEMM, indicating that they exert normal function as multipotent precursors. However, the proportion of erythroid progenitor CFU-E in HSPCs derived from OGTi–C11 hiPSCs was significantly higher than that from pLenti hiPSCs, while its CFU-M was lower than other groups, indicating that inhibition of OGT in hiPSCs yields HSPCs with erythroid bias in our system. In addition, the derived HSPCs can be differentiated into CD3^+^ CD56^−^ TCR⍺/β^+^ T cells, thus confirming their lymphoid differentiation potential.

## Discussion

*O*-GlcNAcylation, mediated by the cycling enzymes OGT and OGA, contributes to the regulation of hematopoiesis, which produces and replenishes various cell types comprising the blood system, by means of HSC fate decision and lineage specification (Zhang et al., 2019; Luanpitpong et al., 2021; Luanpitpong et al., 2022a; Luanpitpong et al., 2022b). Recent studies have demonstrated that *O*-GlcNAcylation also affects the functions of red blood cells and various immune cells. Inhibition of OGA in erythroblasts, which subsequently causes hyper-*O*-GlcNAcylation, regulates β- and α-globin production via BCL11A, affecting its oxygen-carrying property (Luanpitpong et al., 2022a). During T cell activation, *O*-GlcNAcylation is increased, as it is required for the activation of many involved transcription factors, such as NFAT, c-Rel and c-Myc (Golks et al., 2007; Chang et al., 2020). Challenges in studying hematopoiesis using HSCs, the most primitive cell type in the hierarchy, as a study model are their relatively poor susceptibility to genetic modification and the limited extent to which they can be expanded *ex vivo* (Becker et al., 2023; Buffa et al., 2023). If the genes of interest are essential for HSC survival and/or stemness, it is unlikely to maintain long-term culture of functional HSCs. We postulated that *OGA* and *OGT* are essential genes in HSCs, as we could not obtain viable *OGA*- and *OGT*-depleted CD34^+^ HSPCs in our tested system (Luanpitpong et al., 2022b). Notably, although we could establish transient *OGA*- and *OGT*-depleted LSCs, we could never obtain the stable clones, as they could not be clonally expanded (Luanpitpong et al., 2022b). To support this notion, mouse *Ogt* deletion is embryonic lethal, and *Oga* deletion is perinatal lethal (Shafi et al., 2000; Muha et al., 2021). In zebrafish, Ogt and Oga overexpression were both shown to disrupt T-cell survival and cause delays in epiboly (Webster et al., 2009).

Recently, hiPSCs have drawn attention in biomedical research as a tool for modeling hematopoiesis and hematologic disorders. Herein, isogenic, single-cell clones of OGAi (C17) and OGTi (C11) hiPSCs generated by CRISPR/Cas9 with approximately 80% editing efficiency and validated INDEL mutations were obtained along with their counterpart control pLenti hiPSCs (Figures 1, 2). The established OGAi–C17 and OGTi–C11 hiPSCs, which revealed minimal CRISPR/Cas9 off-target potential, were registered in hPSCreg^®^ as MUSIi012-A-7 and MUSIi012-A-8, respectively. Successful manipulation of cellular *O*-GlcNAcylation in the isogenic pairs that share the same genetic background was shown—OGA inhibition in hiPSCs resulted in a substantial increase in cellular *O*-GlcNAcylation, while OGT inhibition had an opposite effect (Figure 3). Hence, these hiPSC cell lines can be used as a tool to define the functional roles of *O*-GlcNAcylation and its downstream signaling pathways in various aspects and at different hierarchical levels of HSC development. As shown in the present study, they could be used to investigate the roles of *O*-GlcNAcylation in hiPSC differentiation toward HSPCs. A better understanding of the key mechanisms controlling HSPC derivation, maintenance, and expansion would enable the efficient *ex vivo* production of HSPCs and, ultimately, lead to the translation to clinical applications. Currently, HSC therapy in the form of allogeneic and autologous HSC transplantation has been used in a broad range of hematologic disorders, including hematologic malignancies, but its major barrier is the limited cell number (Rubio-Lara et al., 2023). Our findings demonstrate for the first time that inhibition of OGA in hiPSCs improves the yield of HSPCs by approximately 3-fold and the yield of CD235a^+^ erythroid cells by approximately 6-fold when compared with those derived from control hiPSCs under the same conditions (Figure 7).

Once we obtained differentiated HSPCs, they can be used to further determine the roles of *O*-GlcNAcylation in later stages of hematopoiesis, i.e., during the lineage-specific differentiation into various terminally differentiated blood cells. CFU assay revealed that inhibition of OGT in hiPSCs yielded HSPCs with a greater proportion of CFU-E in our system, indicating its erythroid-bias differentiation potential (Figure 8). Consistently, we previously reported that inhibition of OGT in CD34^+^ HSPCs enriched from umbilical cord blood and G-CSF-mobilized peripheral blood by a small molecule inhibitor OSMI-1 promoted the differentiation of HSPCs into erythroid progenitor BFU-E (Luanpitpong et al., 2022a; Luanpitpong et al., 2022b), thus strengthening that our established OGAi–C17 and OGTi–C11 hiPSCs are suitable models for studying hematopoiesis.

Autologous or allogeneic HSCs collected from various sources and hiPSCs have been listed as reliable cell sources for generation of CAR-immune cell-based therapeutics (Arias et al., 2021). Multiple clinical trials have tested CAR-NK cells from *ex vivo* differentiation of CAR-engineered hiPSCs for the treatment of hematologic malignancies (NCT04245722; NCT05182073; NCT04023071) and solid cancers (NCT04106167; NCT04551885). Although how *O*-GlcNAcylation affects NK cell differentiation remains to be studied, enhanced *O*-GlcNAcylation seems to reduce the cytotoxic activity of NK cells (Chang et al., 2020; Feinberg et al., 2022). To our knowledge, the roles of *O*-GlcNAcylation in immune cells are not that simple—loss of homeostasis in *O*-GlcNAcylation, instead of a simple one-way increase or decrease in its level, is shown to be a critical integrator. For example, both an increase and a decrease in *O*-GlcNAcylation resulted in a similar hyperinflammatory response in macrophages (Li et al., 2017; Li et al., 2019) and the promotion of myeloid differentiation in CD34^+^ HSPCs (Luanpitpong et al., 2022b). Currently, relatively little is known about the significant roles of *O*-GlcNAcylation and underlying *O*-GlcNAc signaling in various lineages of immune cells in great detail, as often we could not obtain certain types of progenitors and immature cells for *in vitro* culture. The established OGAi–C17 and OGTi–C11 hiPSCs would help us to fill the current gap. One of the strengths of our hiPSC platform is the incomplete knockout of *OGA* and *OGT* expression; therefore, the OGA and OGT levels can still be inducible or repressible, i.e., by small molecules, allowing molecular dynamics studies of *O*-GlcNAcylation. A limitation of the present study is the lack of analysis of more than one clone per hiPSC line. Due to the technical challenges in keeping the manipulated hiPSCs alive, undifferentiated, and genetically stable, we are currently in the process of establishing more stable clones to improve our platform for further studies.

In summary, it remains to be evaluated whether modulation of cellular *O*-GlcNAcylation via its cycling enzymes OGA and OGT affects hematopoiesis at various stages and lineages of blood cells. The established isogenic OGAi–C17 and OGTi–C11 hiPSCs can be utilized as an experimental platform to shed light on the roles of *O*-GlcNAcylation in normal hematopoiesis and pathogenesis of hematologic disorders. Additionally, these hiPSC lines can be applied to modeling other diseases, e.g., cancers, diabetes, neurodegenerative diseases, and cardiovascular diseases, whose etiology are associated with aberrant *O*-GlcNAcylation (Dias and Hart, 2007; de Queiroz et al., 2014; Bolanle et al., 2021).

## Data Availability

The datasets presented in this study can be found in online repositories. The names of the repository/repositories and accession number(s) can be found below: https://www.ncbi.nlm.nih.gov/genbank/, PP104941 https://www.ncbi.nlm.nih.gov/genbank/, PP104942 https://www.ncbi.nlm.nih.gov/genbank/, PP104943 https://www.ncbi.nlm.nih.gov/genbank/, PP104944 https://www.ncbi.nlm.nih.gov/genbank/, PP104947 https://www.ncbi.nlm.nih.gov/genbank/, PP104948.
