# Supplementary material for: OGT and OGA gene-edited human induced pluripotent stem cells for dissecting the functional roles of O-GlcNAcylation in hematopoiesis
Source: Front Cell Dev Biol. 2024 May 1;12:1361943. doi: 10.3389/fcell.2024.1361943 (PMC11094211; doi:10.3389/fcell.2024.1361943)
Supplement: Supplementary file 1 [file DataSheet1.PDF]

Supplementary Material

OGT and *MGEA5* gene-edited human induced pluripotent stem cells for dissecting the functional roles of *O*-GlcNAcylation in hematopoiesis

Sudjit Luanpitpong*, Kantpitchar Tangkiettrakul, Xing Kang, Pimonwan Srisook, Jirarat Poohadsuan, Parinya Samart, Phatchanat Klaihmon, Montira Janan, Chanchao Lorthongpanich, Chuti Laowtammathron, Surapol Issaragrisil

*** Correspondence:**
Sudjit Luanpitpong
sudjit.lua@mahidol.edu

Supplementary Table 1. Key resources table.

**Reagents**	**Source**	**Catalog number**
hiPSC culture		
NutriStem XF medium	Sartorius	05-100-1A
Matrigel	Corning	356234
Versene	Gibco	15040066
Spontaneous differentiation (EB formation)		
Dispase	Gibco	17105-041
KnockOut™ DMEM	Gibco	10829018
KnockOut™ Serum Replacement	Gibco	10828028
MEM non-essential amino acid	Gibco	11140050
β-mercaptoethanol	Gibco	21985-023
Insulin-transferrin-selenium-ethanolamine	Gibco	51500056
Penicillin/Streptomycin	Gibco	15140-122
HSC differentiation		
StemPro™-34 SFM	Gibco	10640-019
GlutaMAX™	Gibco	25030081
Accutase™	Stemcell technologies	07920
Human transferrin	Roche	10652202001
Monothiolycerol (MTG)	Sigma-Aldrich	M6145
Rho Kinase inhibitor (ROCK) Y27632	Abcam	ab120129
L-ascorbic acid	Sigma-Aldrich	A4403-100MG
rh BMP4	Miltenyi Biotec	130-098-786
rh BFGF	Peprotech	100-18B

CHIR99021	Tocris	4423
rh VEGF	R&D Systems	293-VE/CF
rh IL-3	R&D Systems	203-IL
rh IL-6	R&D Systems	206-IL
rh IL-11	Miltenyi Biotec	130-103-439
rh IGF-1	R&D Systems	291-G1
rh EPO (EPREX®)	Janssen-Cilag AG	-
rh TPO	R&D Systems	288-TP
rh FLT-3L	Miltenyi Biotec	130-096-474
rh SCF	R&D Systems	255-SC
CFU assay		
MethoCult™ H4435 Enriched	STEMCELL Technologies	04435
Antibiotics and compound		
Hexadimethrine bromide	Sigma-Aldrich	H9268
Ampicillin	Sigma-Aldrich	A9393
Puromycin dihydrochloride	Sigma-Aldrich	P7255
Antibodies for Western blotting (dilution)		
Anti-β-Actin (8H10D10) (1:5000)	Sigma-Aldrich	A3854
Anti-O-GlcNAc (O-Linked N-Acetyl-glucosamine) (RL2) (1:1000)	Abcam	ab2739
Anti-MGEA5/OGA (EPR7154(B)) (1:1000)	Abcam	ab124807
Anti-OGT / O-Linked N-Acetyl-glucosamine Transferase (1:1000)	Abcam	ab96718
HRP-conjugated anti-mouse (1:5000)	Merck Millipore	AP124P
HRP-conjugated anti-rabbit (1:5000)	Merck Millipore	AP132P
Antibodies for immunofluorescence staining (dilution)		
Rabbit anti-OCT4 (1:300)	Santa Cruz Biotechnology	SC-9081
Rabbit anti-NANOG (1:300)	Thermo Fisher Scientific	PA1-097
Rabbit anti-SOX2 (1:300)	Millipore	AB5603
Alexa Fluor 488 Goat anti-rabbit IgG (1:500)	Thermo Fisher Scientific	A11008
Alexa Fluor 488 Goat anti-mouse IgG (1:500)	Thermo Fisher Scientific	A11001
Antibodies for flow cytometry (dilution)		
FITC-conjugated SSEA-3 (1:50)	BioLegend	330306
PE-conjugated SSEA-4 (1:50)	BioLegend	330406
Alexa Fluor 647 anti-human TRA-1-60 (1:50)	BioLegend	330606
FITC-conjugated CD34 (1:20)	BioLegend	343504
PerCP-conjugated CD45 (1:20)	BD Biosciences	347464
PE-Cy7-conjugated CD43 (1:20)	BioLegend	343208
APC-conjugated CD235a (1:20)	BioLegend	349114

Cocktails and kits		
cOmplete^{TM} Protease Inhibitor Cocktails	Roche Diagnostics	11697498001
Cell Lysis Buffer (10X)	Cell Signaling Technology	9803
Pierce BCA protein assay kit	Thermo Fisher Scientific	23225
Enhance chemiluminescence detection system	Merck Millipore	WBKLS0500
Power SYBR^{TM} Green PCR Master mix	Thermo Fisher Scientific	4472908
RevertAid First Strand cDNA Synthesis Kit	Thermo Fisher Scientific	K1622
BD Trucount^{TM} Tubes	BD Biosciences	340334
PureLink Genomic DNA mini kit	Invitrogen	K1820-02
Q5 High-fidelity DNA polymerase	New England Biolabs	M0491S
GenepHlow/Gel PCR kit	GeneAid	DFH100

Supplementary Table 2. List of primers used for qPCR.

Target	Forward / reverse primer (5'−3')
OCT4	GAAGTTAGGTGGGCAGCTTG / TGTGGCCCCAAGGAATAGT
NANOG	GAGATGCCTCACACGGAGAC / AGGGCTGTCCTGAATAAGCA
SOX2	GGGGGAATGGACCTTGTATAG / GCAAAGCTCCTACCGTACCA
TUBB3	GTCAGACACGGTGGTGGAG / CGATACCAGGTGGTTGAG GT
MAP2	GGAGAGAAAAGGGACTCCA / TATCGGAAGCCAGAGGAGAA
RBFOX3	CAACGGCTGGAAGCTAAATC / CGCAGCCCGAAATGTATTAT
OTX1	GTCTTGTGAGCCCAGGAATG / GCAGGCAATGGACATCTTTT
HAND1	AAGGCTCAGGACCCAAGAAG / GTCCATCAGGTAGGCGATGT
TBX6	AGGCCCGCTACTTGTTTCTT / GTGAGCTTGACACGATGGAA
ACTA2	TATCAGGGGGCACCACTATG / GCTGGAAGGTGGACAGAGAG
NKX2-5	GGTGGAGCTGGAGAAGACAG / AGATCTTGACCTGCGTGGAC
TBXT	TGCTTCCCTGAGACCCAGTT / GATCACTTCTTTCCTTTGCATCAAG
SOX17	ACTACCGCGACTGCCAGAGT / CCAGCGTAGTCCGAGACCT
LEFTY1	AACTTCTGGCAGCAGCTGAG / AAGGTCCAGGGTGTGCAG
AFP	GCTGGAACGTGGTCAATGTA / CTGAGACAGCAAGCTGAGGA
PDX1	GCACCTTCACCACCACCT / CACGCGTGAGCTTTGGTAG
FOXA2	CGTTCCGGGTCTGAACTG / ACCGCTCCCAGCATACTTT
GADPH	AGCCACATCGCTCAGACAC / GCCCAATACGACCAAATCC

Supplementary Table 3. Number of multiple types of progenitor cell colonies in the CFU assay of hiPSC-derived HSPCs.

Number of colonies / CFU types	hiPSC-derived HSPCs		
	pLenti	OGAi	OGTi
CFU-E	11.83 ± 4.49	11.67 ± 2.80	14.00 ± 2.83
BFU-E	1.33 ± 1.51	0.50 ± 0.55	0.50 ± 0.84
CFU-GM	24.00 ± 8.53	13.83 ± 7.25	12.17 ± 2.48
CFU-M	5.60 ± 2.06	3.67 ± 0.82	1.67 ± 0.52
CFU-GEMM	1.17 ± 1.33	0.33 ± 0.52	0.17 ± 0.41

Colonies were visualized and scored under an inverted microscope at 14 days of culture by two certified medical technicians. Data are means ± s.d. (n = 6) from six independent experiments.

A

OGA gRNA #1

Coordinates	Strand	MM	Target sequence	PAM	Gene name	% Eff.
chr10:101803790-812	+	0	GGTGTGGATAGCAACGTAGT	TGG	OGA	67
chr8: 20436442−464	+	5	ATTCTTGATACCAACGTAGT	AGG	RP11-563N12.2	0.8
chr5:178826723−745	−	5	CGTTTTAATAGCTACGTAGT	AGG	RP11-21I4.3	3.6
chr2:153644719−741	−	5	GGTTGGAATTCCAACGTAGT	AGG	AC012501.2	3.3
chr2:25729877−899	+	5	CGTGAGTTTAGCGACGTAGT	AGG	ASXL2	0
chr13:89883292−314	+	5	ACTTTTGATAGCAATGTAGT	GGG	LRRC8D	0.5

OGA gRNA #3

Coordinates	Strand	MM	Target sequence	PAM	Gene name	% Eff.
chr10:101810189−211	−	0	ATCCACATTGAAACGTAAAT	TGG	OGA	73
chr8:11906784−806	+	4	GCCAAAATTGAAACGTAAAT	AGG	RP110589N15.1	2.1
chr9:85096334−56	−	4	ATGGATATGGAAACGTAAAT	GGG	UBE2V1P10	1.7
chr8:1121367296−318	+	4	ATAAAAATTGTAACGTAAAT	GGG	RPL35AP19	0.4
chr1:167974982−5004	−	4	TTCCATAATGACACGTAAAT	TGG	DCAF6	2.1
chr14:73651314−36	+	3	ATCCATGTTGAAAAGTAAAT	TGG	DNAL1	2.2

B

OGT gRNA #3

Coordinates	Strand	MM	Target sequence	PAM	Gene name	% Eff.
chrX:71544602−24	+	0	GCTCAAAGCCCTGGGTCGCT	TGG	OGT	89
chr14:100839253−75	+	4	TGGAAAAGCCCTGGGTCGCT	GGG	MEG3	4
chr22:20062121−43	−	4	GCACTCAGCCCAGGGTCGCT	GGG	TANGO2	7.2
chr7:47239386−408	−	4	CCCCAAATCCCAGGGTCGCT	GGG	TNS3	2.9
chr19:15379364−86	+	4	GTGCAAACCCCGGGGTCGCT	GGG	AC005785.2	2.1
chr3:32503497−519	+	3	GCCCAAAGGCCTGTGTCGCT	AGG	CMTM6	0.9

Supplementary Figure 1. DNA sequencing and ICE analysis of off-target sites in OGAi and OGTi hiPSCs. Potential off-target sites were predicted by CCTop tool. Summary of editing efficient (% Eff.) determined by ICE in the DNA sequencing spanning over Cas9 cut sites on off-target sites in OGAi –

C17 (A) and OGTi – C11 (B) hiPSCs when using corresponding gRNA against *OGA* and *OGT*, respectively.

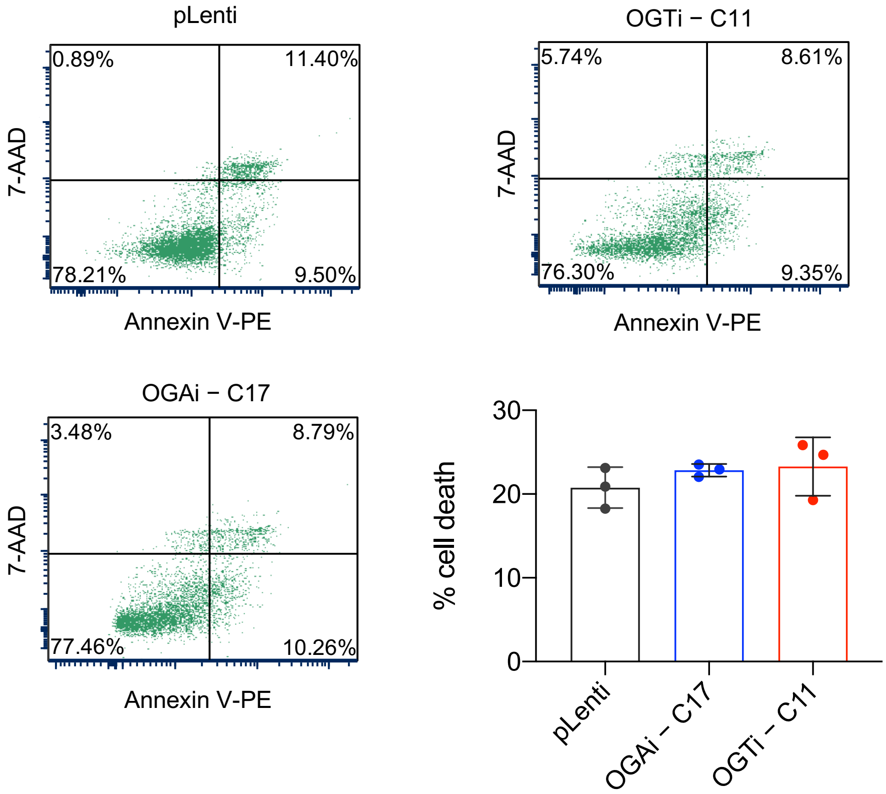

Supplementary Figure 2. Analysis of cell death in OGAi and OGTi hiPSCs by Annexin V/7-AAD assay. Percentages of cell death comprising Annexin V- and/or 7-AAD-positive cells were plotted. Data are mean ± SD (n = 3).

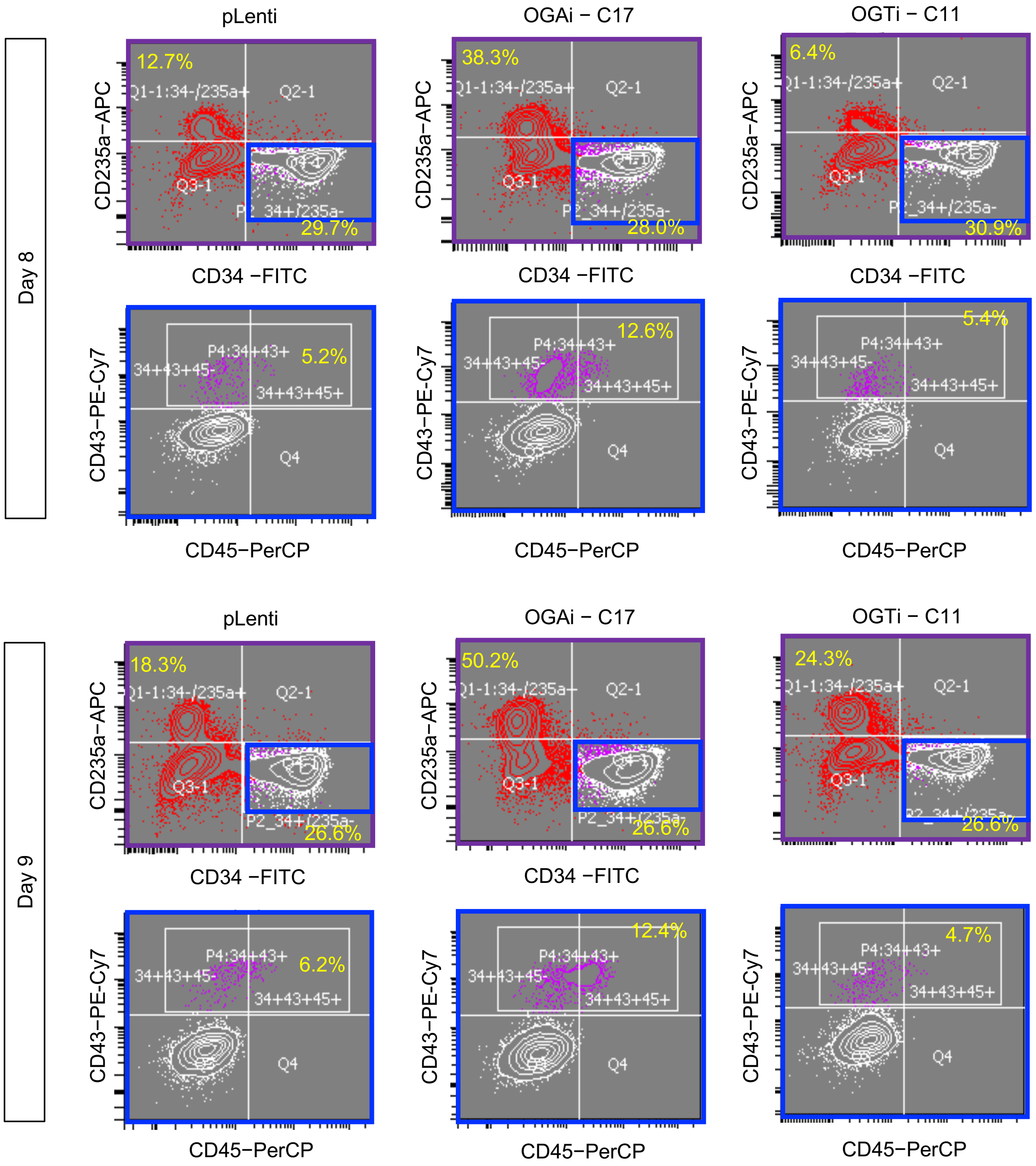

Supplementary Figure 3. Representative flow cytometric plots in correspond to the data of hiPSC differentiation toward HSPCs on days 8 and 9 of culture in Figure 7.

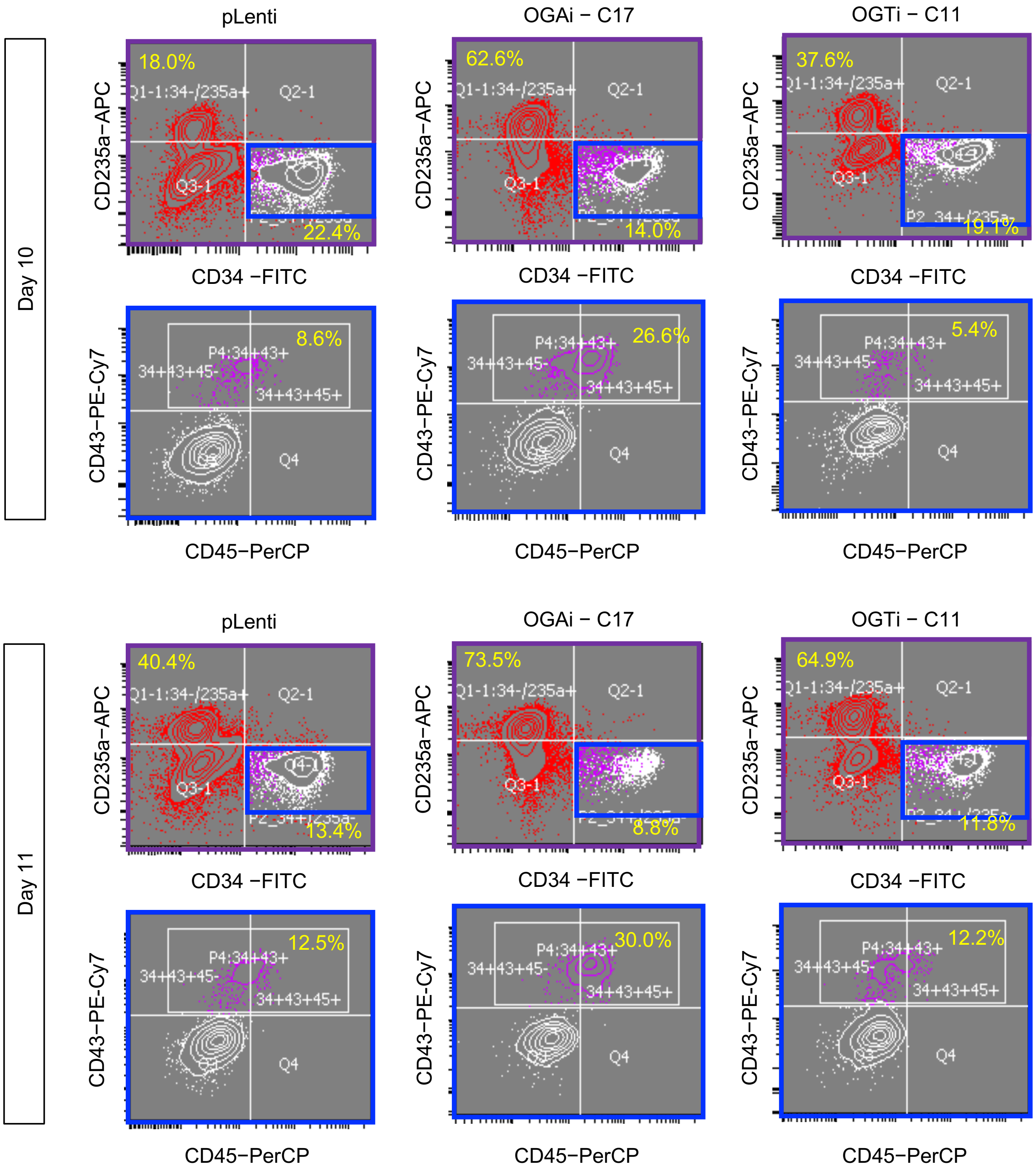

Supplementary Figure 4. Representative flow cytometric plots in correspond to the data of hiPSC differentiation toward HSPCs on days 10 and 11 of culture in Figure 7.

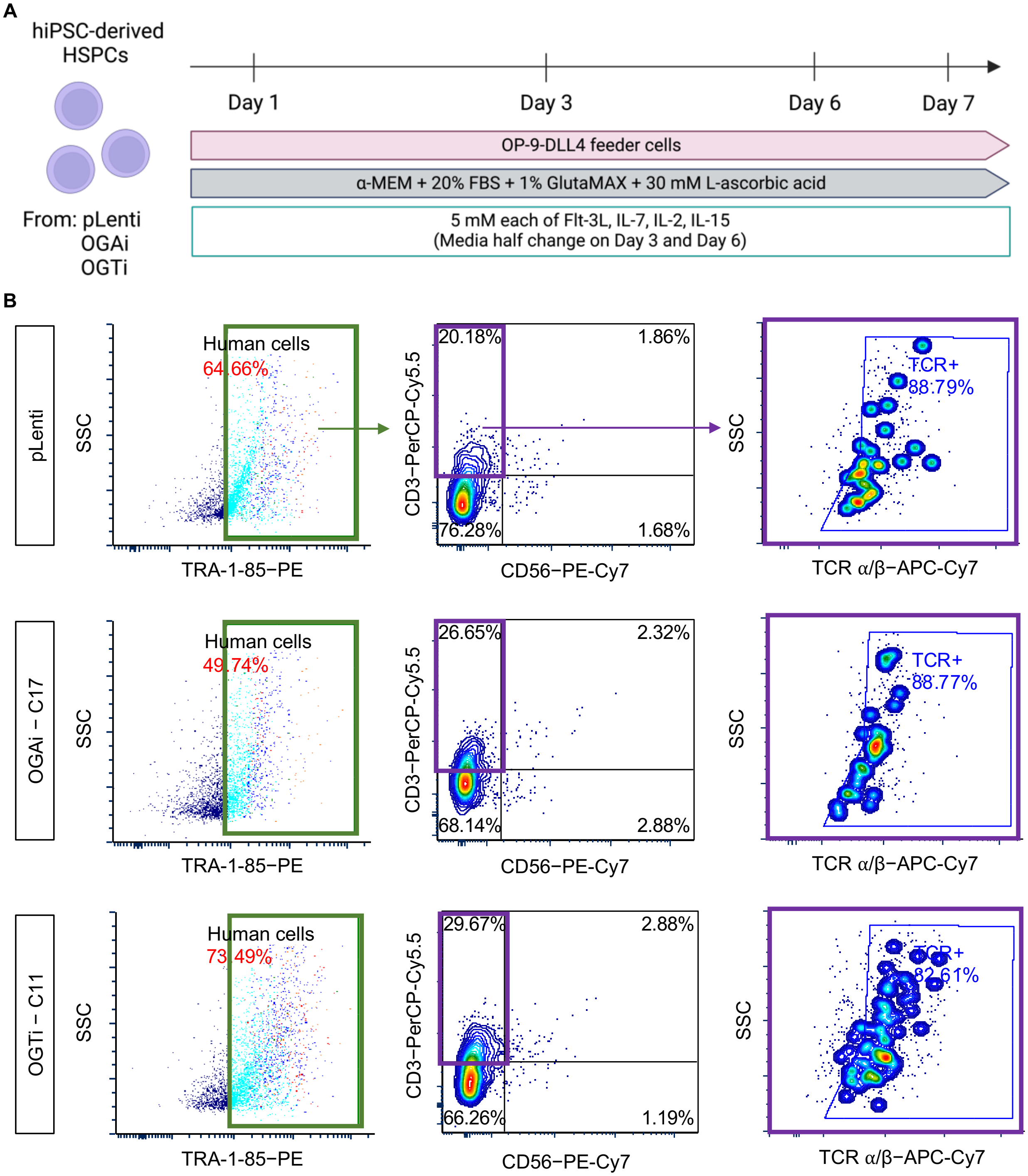

Supplementary Figure 5. T cell differentiation of the differentiated HSPCs from hiPSCs. (A) Schematic diagram showing the differentiation protocol using mouse OP9-DLL4 as feeder cells. HSPCs derived from pLenti, OGAi – C17, and OGTi – C11 hiPSCs at day 11 of culture were used. (B) Representative flow cytometry plots of derived T cells, as defined by CD3^{+} CD56^{-} TCRα/β^{+}, on day 7 of culture.
